# Interaction between *Metarhizium anisopliae* and Its Host, the Subterranean Termite *Coptotermes curvignathus* during the Infection Process

**DOI:** 10.3390/biology10040263

**Published:** 2021-03-25

**Authors:** Samsuddin Ahmad Syazwan, Shiou Yih Lee, Ahmad Said Sajap, Wei Hong Lau, Dzolkhifli Omar, Rozi Mohamed

**Affiliations:** 1 Department of Forest Science and Biodiversity, Faculty of Forestry and Environment, Universiti Putra Malaysia, Serdang 43400, Malaysia; gs50910@student.upm.edu.my (S.A.S.); leesyih@hotmail.com (S.Y.L.); ahmadsaid51@gmail.com (A.S.S.); 2 Mycology and Pathology Branch, Forest Biodiversity Division, Forest Research Institute Malaysia (FRIM), Kepong 52109, Malaysia; 3 Department of Plant Protection, Faculty of Agriculture, Universiti Putra Malaysia, Serdang 43400, Malaysia; lauweih@upm.edu.my (W.H.L.); zolkifli@upm.edu.my (D.O.)

**Keywords:** biological control, entomopathogenic fungi, SEM, TEM, termite proteome, host–pathogen interaction

## Abstract

**Simple Summary:**

The use of *Metarhizium anisopliae* as a biological control of insect pests has been experimented in the laboratory as well as in field trials. This includes against the termite *Coptotermes curvignathus,* however the results have varying degrees of success. One reason could be due to the lack of detailed knowledge on the molecular pathogenesis of *M. anisopliae.* In the current study, the conidial suspension of *M. anisopliae* isolate PR1 was first inoculated on the *C. curvignathus,* after which the pathogenesis was examined using two different approaches: electron microscopy and protein expression. At the initiation stage, the progression observed and documented including adhesion, germination, and penetration of the fungus on the cuticle within 24 h after inoculation. Later, this was followed by colonization and spreading of the fungus at the cellular level. Proteomics of *C. curvignathus* witnessed the expression of proteins related to cell regulation and defense, while in *M. anisopliae*, protein related to transport and fungal virulence were expressed throughout the infection. These findings offer relevant knowledge for use in the development of *M. anisopliae* as a prospective biological control agent for termites in the future.

**Abstract:**

*Metarhizium anisopliae* (Metchnikoff) Sorokin, a pathogenic fungus to insects, infects the subterranean termite, *Coptotermes curvignathus* Holmgren, a devastating pest of plantation trees in the tropics. Electron microscopy and proteomics were used to investigate the infection and developmental process of *M. anisopliae* in *C. curvignathus*. Fungal infection was initiated by germ tube penetration through the host’s cuticle as observed at 6 h post-inoculation (PI), after which it elongated into the host’s integumental tissue. The colonization process continued as seen from dissemination of blastospores in the hemocoel at 96 h PI. At this time point, the emergent mycelia had mummified the host and forty-eight hours later, new conidia were dispersed on the termites’ body surface. Meanwhile, hyphal bodies were observed in abundance in the intercellular space in the host’s body. The proteomes of the pathogen and host were isolated separately using inoculated termite samples withdrawn at each PI-time point and analyzed in two-dimensional electrophoresis (2-DE) gels. Proteins expressed in termites showed evidence of being related to cell regulation and the immune response, while those expressed in *M. anisopliae*, to transportation and fungal virulence. This study provides new information on the interaction between termites and its entomopathogen, with potential utilization for developing future biopesticide to control the termite population.

## 1. Introduction

*Metarhizium anisopliae* is an entomopathogenic fungus of a wide range of hosts, including subterranean termites and other insect members from the class Insecta [1]. There are several stages in the life cycle of *M. anisopliae* when infecting a host: adhesion, germination, penetration, invasion, colonization, and dissemination [2]. The occurrence of each stage is affected by the fungal isolate, host, and environment conditions [3]. Subterranean termites are social insects [4]; it has been suggested that their behavior could be the reason that stops *M. anisopliae* from spreading to other healthy members such as alarming by vibration and aggregation, grooming, defecating, and burying the infected colonies member [5,6,7,8]. These actions are commonly not taken into consideration during in vitro pathogenicity studies because the experimental termites were kept solitary after exposure to fungal spores [9,10,11]. Thus, higher termite mortality is recorded in laboratory experiments compared to field trials. This explains why promising laboratory results were not always reproducible in the field [12,13].

The quest for effective biological control of termites continues with some seeing the ability to use entomopathogens including *M. anisopliae* in addition to chemical insecticides. *Metarhizium anisopliae*, a natural insect pathogen, provides an environmentally friendly alternative to pesticides. Due to its wide range of hosts, this pathogen was used in several termite experiments. The first preliminary screening of the *Coptotermes* genus was performed using two entomopathogens, *M. anisopliae* and *Beauvaria bassiana*, on *Coptotermes formosanus* [14]. *Metarhizium anisopliae* induces higher mortality in this genus than *B. bassiana.*

Insect defense mechanisms were also tested at the molecular level. Through next-generation sequencing technologies, various gene–protein interactions were analyzed and described on *Coptotermes* transcriptome [15]. Several pattern recognition receptors (PPRs) such as β-1,3-glucan recognition proteins (bGRPs), c-type lectins (CTL), down syndrome cell adhesion molecules (DSCAM), hemolin, multidomain scavenger receptors (SCRs), nimrods, thioester-containing proteins (TEPs), galectins (GALE), and fibrinogen-like domain immunolectins (FBNs) have been reported as being active in the defense mechanism on termite transcriptome [15].

The PPRs from another insect host (*Manduca sexta*) were also observed to initiate an immune response by recognizing the present pathogen-associated molecular patterns (PAMPs) such as β-1,3-glucan from the fungi in the host system [16]. Besides, signal modulation protein such as serine protease inhibitors (SPIs) amplifies the pathogen invasion signals once the PAMPs are detected by the PPRs in the *Drosophila* host [17]. Through a series of signaling pathway, for example the c-jun N terminal kinase (JNK) [18]; production of antimicrobial peptides (AMPs) such as defensin, cecropin, metchnikowin, tenascin 1 and tenascin 2 are initiated [19,20]. 

In this host–pathogen interaction, *M. anisopliae* has its own mechanism to invade and spread within the host. Starting from detection of fungal adhesion on the host’s cuticle surface, several proteins have been detected from *M. anisopliae* such as the ATP synthase F1 beta subunit, MAD1 adhesin, and heat shock protein (HSP) [21,22]. MAD1 adhesin protein plays a role in adhesion once the conidia are deployed on the host’s cuticle. The presence of HSP is to boost the survival ability of the conidia when facing environmental stress [23]. Upregulation of chitinase and protease genes correspond with differentiation of appressoria [24,25]. Colonization of the insect host continues with production of fungal propagules and toxins such as destruxins [26], which can kill the host. 

While many findings about the fungal reaction challenging insect host, little is known on the termite’s defense reaction against fungal pathogen in vitro. In the present study, we used scanning electron microscope (SEM) and transmission electron microscope (TEM) techniques to investigate pathogenesis caused by *M. anisopliae* because the pathogen appeared to be highly lethal, causing death to *C. curvignathus* within 36–48 h post-inoculation (PI) [13]. Furthermore, we analyzed the proteomes of the termite and pathogen as the infection was in progress. Our results provide new knowledge on the defense and immunity responses of *C. curvignathus* in keeping *M. anisopliae* threats at bay. This will enable the development of management strategies for controlling *Coptotermes* subterranean termites in the future.

## 2. Materials and Methods

### 2.1. Termite Collection

Termites were collected from their natural nest located at the Expo Hill, Universiti Putra Malaysia (UPM), Serdang, Selangor, Malaysia. They were trapped using a 5 L cuboid-shaped plastic container with holes of 1 cm in diameter, containing stacks of 20 cm × 6 cm × 2 cm pine wooden blocks (*Pinus caribaea*), placed underground approximately 0.2 m from the infested tree. After seven days, the trapped termites were brought to the laboratory and separated from the debris following the previous methodology [27]. The termites were kept in a 15 cm in diameter plastic cup containing wet filter paper at 28 °C. Only termite workers were used in this study. Termite identification was based on its morphology [28].

### 2.2. Fungal Culture

Pure culture of *M. anisopliae*, isolate PR1 was obtained from the Forest Entomology Lab, Faculty of Forestry, Universiti Putra Malaysia, due to its high virulence [29]. Fungal culture was first grown on Difco™ Sabaroud Dextrose agar (BD Diagnostics, Franklin Lakes, NJ, USA) plates and incubated at 28 °C for 7 d. Termite workers were inoculated by directly dusting the conidial powder onto the termites’ bodies. Then, they were kept individually in 1.5 cm in diameter plastic cups containing moist filter papers (Machery-Nagel GmbH & Co. KG, Düren, Germany). After two weeks, conidia that emerged from the termites’ carcasses were isolated and cultured on Difco™ Sabaroud dextrose agar (BD Diagnostics, Franklin Lakes, NJ, USA) fortified with 1% chloramphenicol and 1% streptomycin sulphate to suppress bacterial growth (Sigma Aldrich Co, St. Louis, MO, USA). The isolated fungus was purified using the optimized single spore method, which was by transferring germinated single conidia into the new media agar aiding with a stereo microscope [30].

### 2.3. Preparation of Conidial Suspension and Inoculation 

Purified fungus was mass-cultured on sterilized rice medium containing 0.1% Bacto™ yeast extract agar (BD Diagnostics, Franklin Lakes, NJ, USA) and kept at 26 °C and 80% relative humidity (RH). After 14 d when the conidia had emerged on the rice medium, the fungal mass was collected and left to dry in a desiccator containing silica gels. Two weeks later, the dried fungal mass was sieved through a 10 microns sieve and the conidia collected. For protein extraction, conidia samples from three different rice medium bags were collected, weighed, snapped-frozen individually in liquid nitrogen, and kept at −80 °C until used.

Conidial suspension was then prepared in sterilized distilled water with 0.05% Tween^®^ 80 (Sigma Aldrich Co, USA) and diluted to 1 × 10^7^ conidia/mL aided by a hemocytometer (Hirchmann Laborgeräte, Eberstadt, Germany). Termites were sterilized by dipping them into 0.5% sodium hypochlorite (commercial bleach), rinsed in sterile distilled water, and then blotted on filter paper to remove excess water. Surface-sterilized termites were then dipped into the conidial suspension for 5 s. Inoculated termites were then transferred individually to a 1.5 cm in a diameter plastic cup and kept at 28 °C and 80% RH. Samples were removed at 1, 3, 6, 12, 24, 48, 96, and 144 h PI, while surface sterilized uninoculated termites were collected at 0 h to serve as a control. For electron microscopy, the termites were transferred into 4% glutaraldehyde (Agar Scientific Ltd., Stansted, Essex, UK) and left at 4 °C overnight for fixation process followed by sample preparation. For protein analysis, three individual inoculated termites representing three biological replicates were collected at each PI time-point resulting with a total of 27 infected specimens. The termites were snapped-frozen individually in liquid nitrogen and kept at −80 °C until used.

### 2.4. Specimen Preparation for Electron Microscopy

The fixed termites were washed three times for 10 min each in 0.1 M sodium cacodylate buffer (Agar Scientific Ltd., Stansted, Essex, UK). The termites were then post-fixed in 1% osmium tetroxide (Agar Scientific Ltd., Stansted, Essex, UK) at 4 °C for 2 h, rewashed in 0.1 M sodium cacodylate buffer, pH 7.4, three times for 10 min each, and dehydrated in a series of acetone: 35, 50, 75, 95, and 100% for 10 min each, except for the final concentration where the samples were dehydrated 3× for 15 min each. The dehydrated specimens were further processed for SEM and TEM specimen preparation at the Microscopy Unit of the Institute of Biosciences, Universiti Putra Malaysia.

Five dehydrated termites were withdrawn at each PI time-point for scanning electron microscopy (SEM) study. Dehydrated termites were transferred into a specimen basket for critical point dryer in a Bal-tec™ CPD 030 critical dryer (Bal-tec™ Inc., Los Angeles, CA, USA). The specimens were mounted onto a stub using double sided tape and were coated with gold (Agar Scientific Ltd., UK) in a Bal-tec™ SCD 005 sputter coater (Bal-tec™ Inc., Los Angeles, CA, USA) and kept in 100 mm × 20 mm glass soda-lime Petri dish in a desiccator with silica gels. The prepared samples were then directly viewed under a JEOL JSM 6400 SEM (JEOL Ltd., Akishima, Tokyo, Japan) at high-vacuum mode.

Meanwhile, dehydrated termites from three time points: 48, 96, and 144 h PI were used for transmission electron microscopy (TEM) study. Five dehydrated termites were withdrawn from the 3 time-points. The dehydrated termites were infiltrated in a series of resin (Agar Scientific Ltd., Stansted, Essex, UK) and acetone at ratios 1:1 for 1 h, 1:3 for 2 h, and only resin, overnight, followed by an additional 2 h before embedding into BEEM^®^ Flat Embedding mold (Ted Pella Inc., Redding, CA, USA). The resin-embedded termite specimens were polymerized at 60 °C for 48 h. Ribbons of semithin sections (1 µm) were cut using a glass knife on a Leica Ultracut UCT Ultramicrotome (Leica Biosystems Nussloch GmbH, Nußloch, Germany). The ribbons were placed on a glass slide and poststained with toluidine blue. Excessive stain was washed out and the stained ribbons were covered with a cover slip and dried on the hot plate. The slide was examined under the light microscope for selecting areas of interest. Once the area of interest was selected, ultrathin sections were made and mounted on a copper grid. Lastly, the sections were stained with uranyl acetate for 15 min and washed two times with double distilled water. The sections were counter stained with lead stain (Electron Microscopy Sciences, Hatfield, PA, USA) for 10 min and again washed two times with double distilled water. The specimens were viewed under a Hitachi H-7100 TEM (Hitachi Ltd., Chiyoda City, Tokyo, Japan). 

### 2.5. Total Protein Extraction, Quantification, and Two-Dimensional Gel Electrophoresis (2-DE)

Protein samples were extracted separately from thirty separately inoculated termites for each time point (1, 3, 6, 12, 24, 48, 96, and 144 h PI), thirty individual uninoculated termites and pure *M. anisopliae* conidia. A total of 100 mg fine powder was obtained from each sample by grinding in liquid nitrogen using mortar and pestle. Total protein was extracted using the ReadyPrep™ Protein Extraction (Total Protein) kit (Bio-Rad, Hercules, CA, USA), following the manufacturer’s protocol. Protein concentration was determined using bovine serum albumin (BSA) standard from Quick Start™ Bradford Protein Assay (Bio-Rad, Hercules, CA, USA), following the manufacturer’s instructions, then quantified using the GENESYS™ 10S UV–Vis Spectrophotometer (Thermo Scientific, Waltham, MA, USA). Prior to two-dimensional gel electrophoresis (2-DE), a total of 100 µg of extracted protein was isolated from the stock extract for the clean-up step using a 2-D Clean-Up kit (Cytiva, Little Chalfont, Amersham, UK), following the manufacturer’s instruction.

Gel electrophoresis was performed using 7 cm Immobiline DryStrip pH 3–10 (GE Healthcare Life Science, UK). Loaded protein suspension was left passively rehydrated for 12 h on IPG strips. The isoelectric focus in the isoelectric focusing (IEF) step was carried out in the Proteomics Laboratory at the Agro-Biotechnology Institute, Malaysia. Isoelectric focusing was performed in the PROTEAN^®^ IEF Cell (Bio-Rad, Hercules, CA, USA) with up to 12,000 Vh at a maximum voltage of 4000 V. Then, the IPG strips were equilibrated using ReadyPrep 2-D Starter Kit Equilibration Buffer I (Bio-Rad, Hercules, CA, USA) and ReadyPrep 2-D Starter Kit Equilibration Buffer II (Bio-Rad, Hercules, CA, USA), following the manufacturer’s instruction. Proteins from DTT/IAA equilibrated IPG strips were then separated on 12% sodium dodecyl sulfate (SDS) polyacrylamide gels; together with Precision Plus Protein™ Dual Color Standards (Bio-Rad, Hercules, CA, USA) using a Mini-PROTEAN^®^ Tetra Cell (Bio-Rad, Hercules, CA, USA) in the chill condition. Gels were stained with Bio-Safe™ Coomassie Stain (Bio-Rad, Hercules, CA, USA) prior to scanning or spot quantification analysis. A total of 30 gels were prepared and analyzed: 3 gels for each time point from different biological replicate samples (1, 3, 6, 12, 24, 48, 96, and 144 h PI) and 2 different control specimens (uninoculated termite and conidia of *M. anisopliae*).

### 2.6. Gel Imaging and Protein Expression Analysis

The gels were scanned using a GS-800™ Calibrated Densitometer (Bio-Rad, USA) and gel images were analyzed using Progenesis SameSpots software (Nonlinear Dynamics, Garth Heads, Newcastle upon Tyne, UK). Scanning of the 2-DE gels was carried out in triplicates with a control (uninoculated healthy termite and conidia of *M. anisopliae*) included for comparisons during each scan. The Progenensis software automatically normalized the volume using the multivariate technique by comparing variation of protein spots expressed on each gel. Results were then filtered with two different parameters: (1) spots expressing more than 2-fold change of expression and (2) *p*-value for expressed protein spot for each time point is *p* < 0.05. Abundance ratio was determined by comparing results from the treated samples to the untreated samples with average relative spot volumes obtained from the samples. Generated results were reviewed and checked before selected for spot excision in downstream application.

### 2.7. In-Gel Digestion and MALDI-TOF Mass Spectrometry (MS) Analysis

Protein spots were excised manually and destained with 50% acetonitrile in 25 mM ammonium bicarbonate (NH_4_HCO_3_) until transparent, then washed with 200 mM NH_4_HCO_3_ to remove the dye. The gel plugs were vacuum-dried, then incubated overnight at 37 °C with 25 µL of 25 mM NH_4_HCO_3_ containing 15 ng/µL trypsin (Agilent Technologies, Santa Clara, CA, USA). The resulting peptides were extracted using 50% and 100% acetonitrile, and subsequently vacuum-dried for MS analysis. Prior to MS analysis, dried peptides were reconstituted in 0.1% formic acid, desalted using ZipTip C18™ pipette tips (Millipore, Burlington, MA, USA) and cocrystallized by adding one volume of the same to one volume of α-hydroxy cinnamic acid (10 mg/mL) after which, 0.7 µL was spotted directly on Opti-Tof 384 well (Applied Biosystem/Thermo Fisher Scientific, Bedford, MA, USA). The peptides were analyzed using the 4800 Plus MALDI TOF/TOF analyzer (Applied Biosystems/Thermo Fisher Scientific, Bedford, MA, USA) located at the Medical Biotechnology Laboratory of the Faculty of Medicine, University of Malaya, with the mass standard kit (Applied Biosystems/Thermo Fisher Scientific, Bedford, MA, USA) as the calibrator for the resulting MS and MS/MS mass spectra scales.

### 2.8. Protein Identification and Annotation

For protein identification of the host and pathogen, peak lists were used and peptide masses were searched using MASCOT version 2.6 (Matrix Science, Boston, MA, USA) against Isoptera and *Metarhizium* spp. entries in the non-redundant protein sequences (NCBInr) database. Common contaminant from cRAP contaminants database was also added. Database search parameters for MASCOT were set as follows: The enzyme trypsin was used; up to one missed cleavage was allowed; variable modification included were carbamidomethylation of cysteine and oxidation of methionine; the peptide tolerance for MS precursor ion and MS/MS fragment ion were 100 ppm and 0.2 Da, respectively; and peptide charge was +1; monoisotopic masses of query and instrument MALDI-TOF-TOF was used in the experiment. The theoretical protein mass and isoelectric point (pI) was estimated by computing the expected peptide sequence on Compute pI/Mw tool online software [31]. The identified proteins were analyzed and categorized based on Cellular Component, Molecular Function and Biological Process by annotating using QuickGo browser [32] against GO database [33].

## 3. Results

### 3.1. Observation of Metarhizium anisopliae Pathogenesis on Inoculated Coptotermes curvignathus

**Figure 1:**
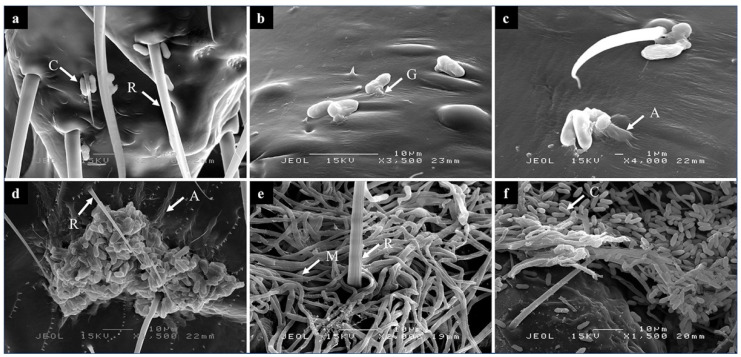
SEM images of *Metarhizium anisopliae* during development of pathogenesis in *Coptotermes curvignathus* at different time-points post-inoculation (PI). (**a**) At 6 h PI, pathogenesis starts with the attachment of fungal conidia near hairs of *C. curvignathus*, (**b**) at 6 h PI, germination of the conidia and penetration through the epicuticle layer, (**c**) at 12 h PI, elongation of appressorium tube along the termites’s exocuticle, and (**d**) at 24 h PI, most of the conidia between the conidial mass attached to the epicuticle layer was germinated and elongated into the integument. (**e**) At 96 h PI, mummification of the termite’s body initiated by elongation of the fungal hypha into mycelium and (**f**) at 144 h PI, emergence of new conidia in abundance on the mycelial mat. R = hair, C = conidia, G = germ tube, A = Appressorium tube, M = mycelium.

Worker termites inoculated with the conidial suspension of *M. anisopliae* were observed using SEM and TEM techniques. Fungal conidia were seen attached to the various parts of the termite’s body including the antennae, thorax, abdomen, and legs. Conidial adhesion happened within 1–3 h PI, evidently near hairs and hair pores (Figure 1a). The pathogenesis process continued with germination of the hyphal tips or germ tubes from the conidia (Figure 1b), which progressed into appressoria as observed at 6 h PI. The appressoria could be seen pressing against the host’s epicuticle at this stage. At 12 h PI, the germ tube elongated and penetrated integument of the termite indicating invasion begins at this hour (Figure 1c). The progress of this pathogen in colonizing the host seems encouraging due to the majority of conidia between the conidial mass found between the hair sockets and the pores on the termite epicuticles was germinated and elongated into the integument within 24 h PI (Figure 1d). In this figure, a significant number of conidia found on the cuticle of termite might have been by chance during the inoculation process, not generally occurring in the pathogenesis of the inoculated fungus. Then, late pathogenesis occurred when the termite became mummified with mycelia in preparation for the formation of conidiogenous cells on the termite’s carcass as seen at 96 h PI (Figure 1e). Finally, new conidia emerged from the mycelial bodies on the mummified termite at 144 h PI (Figure 1f). These are proofs that *M. anisopliae* had succeeded in infecting and colonizing *C. curvignathus*. The average number of days taken by *M. anisopliae* before conidial emergence is approximately 7 days PI [34]. The present study revealed that this could happen in 6 days PI.

**Figure 2:**
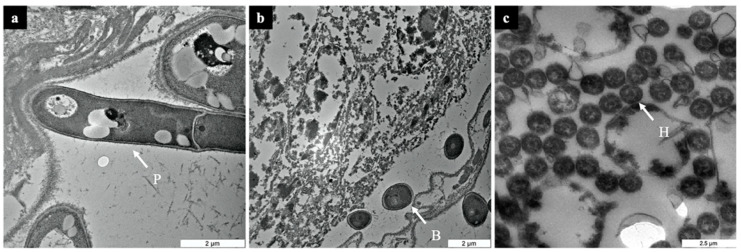
Cross-section images of *Coptotermes curvignathus* after inoculation with *Metarhizium anisopliae* as visualized under TEM. (**a**) 48 h post-inoculation (PI) = a hypha penetrating into the host by pressing on the termite’s epidermal cell, (**b**) 96 h PI = dissemination of blastospores within the hemocoel, and (**c**) 144 h PI = hyphal bodies colonizing the intercellular part of the termite. P = penetrant hypha, B = blastospore, H = hyphal body.

*Metarhizium anisopliae*’s development within the host started at 48 h PI. At this stage, penetration by germinated conidia was successful on the epicuticle of the termite. At 48 h PI, a penetrant hypha (Figure 2a) that had earlier elongated from an appressorium on the termite’s epicuticle (as observed at 6 h PI in Figure 1b), continued to press against the epidermal cell of the termite to gain entry (Figure 2a). Besides providing a major site for adhesion, the appresorium plays a role in mechanical pressuring to pierce the infected host [35,36]. During the invasion at 96 h PI, blastospores are seen being dispersed throughout the hemocoel (Figure 2b). Successful *M. anisopliae* colonization inside the termite’s body is indicated by the presence of a large number of hyphal bodies in the intercellular region of the termite as observed at 144 h PI (Figure 2c).

### 3.2. Protein Identities in the Coptotermes curvignathus–Metarhizium anisopliae Relationship

Total proteins were prepared from 24 individual termites representing three biological replicates from each time-point after inoculation at 1, 3, 6, 12, 24, 48, 96, and 144 h. In addition, total proteins were also extracted from three uninoculated termites and three replicates of the pathogen’s mycelial mass for comparison purposes. Each of the protein sample was separated via 2-DE resulting in a total of 30 gel images with protein spots of various intensities. The 2-DE gel images of the infected termites were compared to that of untreated termite and *M. anisopliae* using Progenesis SameSpots software, yielding with a total of 85 and 90 protein spots identified in *C. curvignathus* and *M. anisopliae*, respectively. Each spot intensity (in volumes) on the PI gel, relative to the control gel was computed by the software. From the means of relative spot intensity, only 22 protein spots had significant expression over the course of pathogenesis (ANOVA, *p* < 0.05). The differentially expressed protein spots were excised and identified. Predicted peptides were then compared to known peptides from the Swiss-Prot database and annotated based on homology with predicted protein information from the NCBInr database. Thirteen of the peptide sequences had high homology to known *C. curvignathus* sequences (Table 1), while the rest (nine) to *M. anisopliae* (Table 2).

### 3.3. Gene Ontology (GO) Annotation

**Figure 3:**
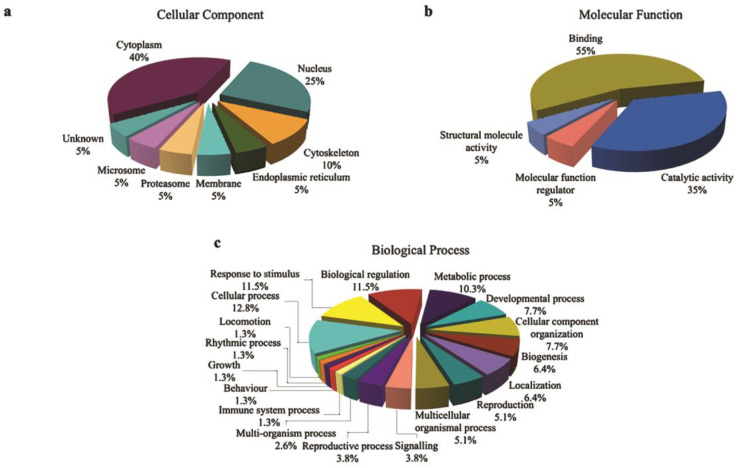
Gene ontology (GO) categories of identified proteins expressed by *Coptotermes* *curvignathus* infected by *Metarhizium* *anisopliae* during pathogenesis development. Identified proteins were annotated and grouped to their respective protein categories: (**a**) cellular component, (**b**) molecular function, and (**c**) biological process using the GO Slim software.

**Figure 4:**
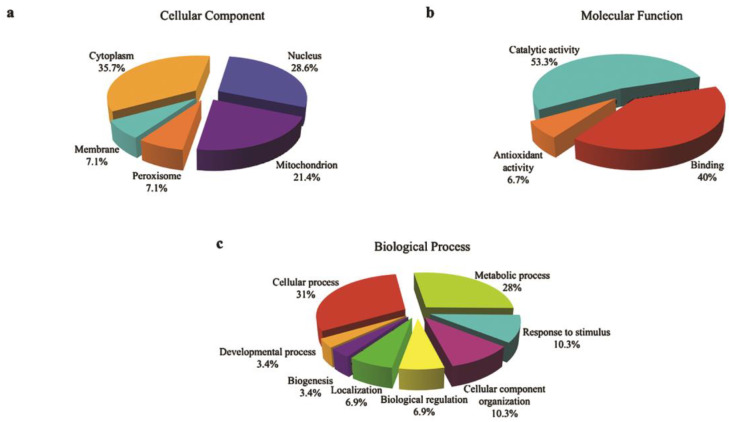
Gene ontology (GO) categories of identified proteins expressed by *Metarhizium* *anisopliae* infecting *Coptotermes* *curvignathus* during pathogenesis development. Identified proteins were annotated and grouped to their respective protein categories: (**a**) cellular component, (**b**) molecular function, and (**c**) biological process using the GO Slim software.

The location and structure of the 22 expressed proteins during pathogenesis was analyzed using GO Slim. The majority of the protein expressed in both species was expressed in the cytoplasm and nucleus. As many as 40% and 25% of the expressed protein by termites (Figure 3a) and 35.7% and 28.6% of the fungus-related proteins (Figure 4a) were expressed in the cytoplasm and nucleus, respectively.

Two major activities belonging to the molecular function were identified in both organisms. Of the expressed proteins in termites 55% and 35% had binding and catalytic functions, respectively (Figure 3b), while, for fungus, 53.3% of its expressed proteins were involved in catalytic activity and the other 40% for binding purposes (Figure 4b). In addition, 6.7% of the identified fungal proteins had some involvement in antioxidant activity that might be expressed due to an interaction with the host’s immune system.

Several of the annotated expressed termite proteins were involved in pathogenesis and the defense mechanism such as a response to the stimulus (11.5%), signaling (3.8%), multiorganism process (2.6%), and immune system process (1.3%), which are useful during colonization and invasion of the infected host (Figure 3c). Furthermore, more than quarter of the fungal expressed proteins had roles in metabolic processes (Figure 4c).

### 3.4. Identified Protein Expressions at Different Stages of Pathogenesis

**Figure 5:**
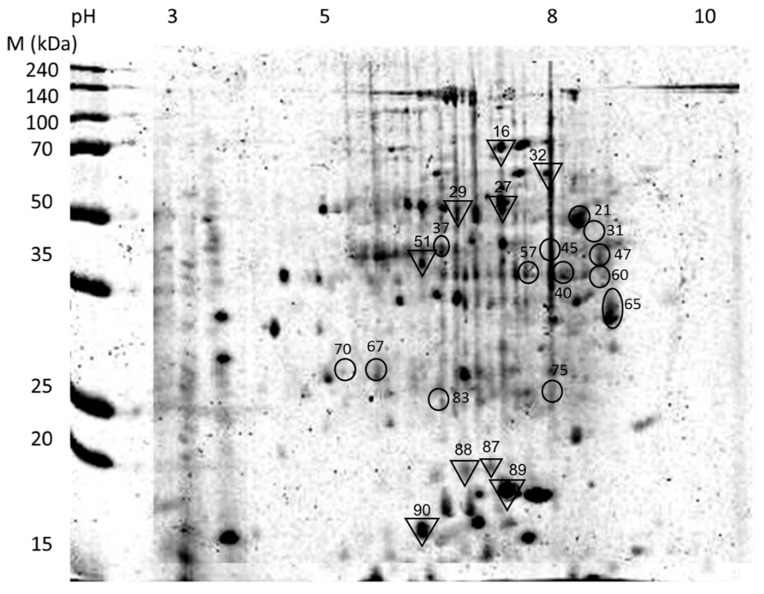
Two-dimensional electrophoresis merged master gel of separated total proteins from the fungus–termite interaction. Protein spots with significant expression are identified in *Coptotermes* *curvignathus* (circles) and *Metarhizium* *anisopliae* (reversed triangles). Numbers shown next to the protein spots are their respective protein IDs.

Analysis on the 2-DE gels using Progenesis Samespots further revealed expression profiles of several of the homology-based identified proteins. Four out of the 13 proteins from *C. curvignathus* and five out of the nine proteins from *M. anisopliae*, (Figure 5) had regulated pattern of expression at different stages of pathogenesis, starting from initiation of infection till the late of pathogenesis. They had distinct significant expression when compared to the other identified proteins (mean of normalized protein spot intensities over 20 × 10^3^).

**Figure 6:**
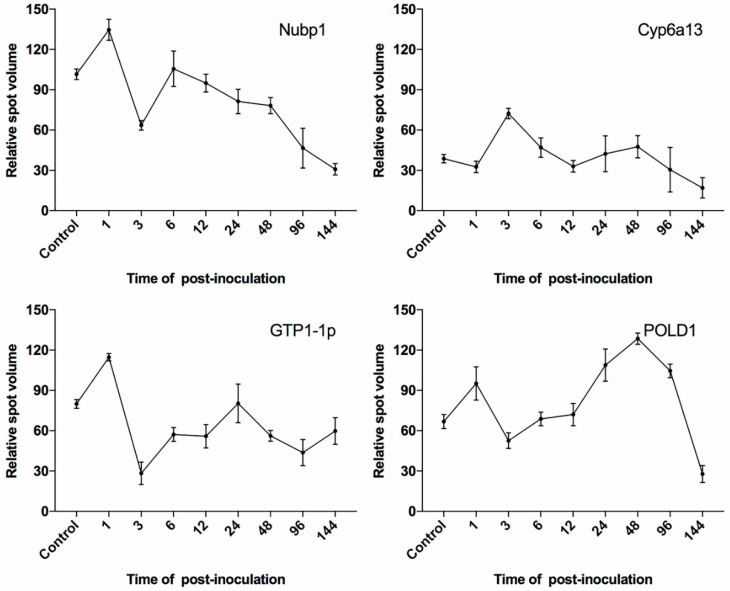
Protein spots of significant expression identified from *Coptotermes* *curvignathus*. *X*-axis: time (hours) of PI; Y-axis: mean of relative spot volume (×10^3^).

Upon protein identification using mass spectrometry, the four proteins (Spot ID: 31, 45, 57, and 65) expressed by *C. curvignathus* were identified as cytochrome P450 6a13 (Cyp6a13), large subunit GTPase 1-like protein (GTP1-lp), cytosolic Fe-S cluster assembly factor Nubp1 (Nubp1), and DNA polymerase delta catalytic subunit (POLD1), respectively (Table 1). The three later proteins were upregulated very early after inoculation (1 h PI), while the Cyp6a13 expression level was upregulated at 3 h PI (Figure 6). However, as the infection progressing, their expressions showed a decreasing pattern, except for POLD1, which increased at 24 h and 48 h, before dropping at 96 h PI. 

**Figure 7:**
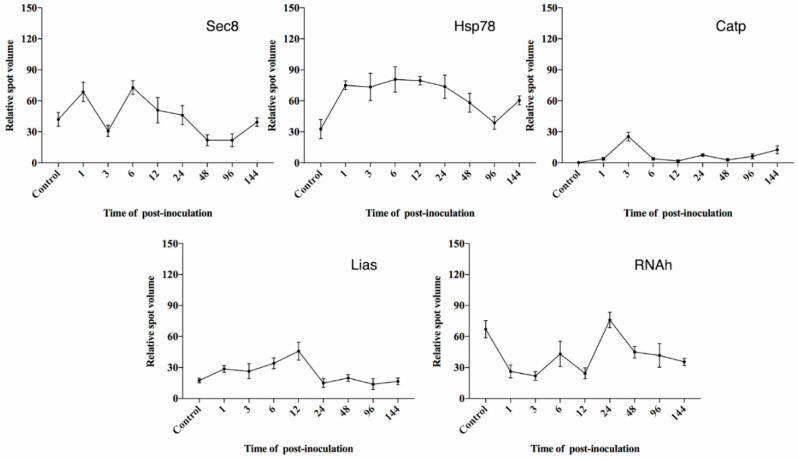
Protein spots of significant expression identified from *Metarhizium* *anisopliae. X*-axis: time (hours) of PI; *Y*-axis: mean of relative spot volume (×10^3^).

Meanwhile, the five proteins spots (Spot ID: 27, 29, 32, 51, and 90) expressed by *M. anisopliae* were identified as Sec8 exocyst complex component specific domain protein (Sec8), lipoic acid synthetase (Lias), heat shock protein 78 (Hsp78), ATP-dependent RNA helicase DBP8 (RNAh), and peroxisomal catalase (Catp), respectively (Table 2). Hsp78 and RNAh had low levels of expression over the course of pathogenesis (Figure 7), but Lias was upregulated until 96 h, and Catp was upregulated twice, at 6 h and 24 h. As for Sec8, it was upregulated at the beginning of the pathogenesis process, at 1 h and 6 h PI, and then downregulated until the end of pathogenesis (Figure 7).

## 4. Discussion

Multiple sites for the *M. anisopliae* conidial attachment were observed on the termite’s epicuticles such as hair sockets and pores. One of the factors that should be accounted for as a successful attack by *M. anisopliae* is the multiple infection sites on the termite’s epicuticles (Figure 1a,b). This allows the fungus to emit the toxin and prevent the chance of successful cellular encapsulation by the termites [37]. Beside this, the unique entomopathogenic fungus develops a mechanical force, which allows the conidia to be attached at several different places. The fungus colonizes the host’s body through penetration using its appressoria on three different sites on the termite’s exoskeleton. A majority of the conidial attachment had occurred mostly on the sternum and in close proximity to the hairs and pores, similar to the plant hopper, *Peregrinus maidis* [38]. Massive mycelial growths along the host’s exocuticle were observed at 24 h PI. At this stage, the fungus had colonized the integumental tissue of the host before entering the hemolymph and reproducing new hyphae and other fungal bodies. The pathogen successfully completed its whole life cycle once it reached the conidiogenesis stage on the termite carcasses in late pathogenesis. This laboratory test provides evidence of the pathogenicity of *M. anisopliae* on *C. curvignathus*, however future experiments are needed to determine if the same efficacy is reproducible in the field.

In natural surroundings, most insects that became host to *M. anisopliae* produce repellent and antifungal compounds for protection, as observed in hosts from the Order of Coleoptera and Orthoptera [39,40], and also in other termite (Order: Blattodea) species, *Reticulitermes flavipes* [41]. In termites, a similar compound known as norharmane is produced by its endosymbionts, which are methanogenic bacteria, *Methanobrevibacter cuticularis* and *M. curvatus* [42,43]. The compound has antifungal activity, which suppresses the mycelial growth of *M. anisopliae* in a non-cellular immune response [41]. Subterranean termites normally build their nests from their wastes, which may contain antifungal compounds produced by the endosymbionts in their guts [37]. This could explain why *M. anisopliae*, a soil-borne fungus, displays very promising results as a biological control agent in laboratory tests but not in field trials [44,45,46]. Organic compounds such as norharmane, can serve as a mobile molecule to mediate interactions between a soil microorganism and fungal pathogens, leading to a boost in the field defense mechanism of termites [47]. In the meantime, in the laboratory, the lack of the structure of the termite nest and the action of surface sterilization on experimental termite with sodium hypochlorite prior to inoculation might lead to the mortality of termites.

Nubp1 was previously described as a scaffold for iron-sulphur (Fe-S) cluster. Biogenesis of FeS requires proteinaceous machineries in both mitochondrion and cytosol [48]. Even though the major function of Nubp1 is as negative regulators of ciliogenesis; a process in which the cell antenna is constructed, it also involves in sensing and processing developmental signals in the cellular level, which deemed essential for membrane trafficking [49]. It is also known to function in cell signaling that regulates the centrosome localization and centriole duplication during cell cycles [50,51]. Nubp1 expression was upregulated at 1 h and 6 h of PI on infected *C. curvignathus*, which may indicate that expression was a part of the response toward the initiation of *M. anisopliae* pathogenesis on the termite’s cuticle. Similarly, GTP1-1p was upregulated at 1 h and 24 h PI. The main function of this protein is its role in GTP binding and GTPase activity. As GTP is an important substrate in signal transduction that is mainly involved with the G-protein couple receptor [52], expression of large subunit GTPase-1 like protein might display relationship with immune cell signaling. This is because GTPase and RHO family, such as Rho GTPase-activating protein 17 (spot 60), is a part of the Ras family that plays a major role in signal transduction pathways during cell proliferation, survival, and apoptosis, which is crucial for effective immune responses [53,54,55]. There is also a claim that it is involved in the complex multiple signaling pathway with another Ras family protein [56].

Cyp6a13 also plays a major role as a defense mechanism action on the cellular level towards infection of *M. aniopliae* on the termites’ host, indicated by upregulation expressed at most time points of the PI stages, which were 3 h, 6 h, 24 h, and 48 h. This protein is well known belonging to the cytochrome P450 family that serves vital roles in insect growth, such as the biosynthesis of hormones, and metabolism of xenobiotics, such as the pesticide or microbial infection [57,58]. It was also reported that this protein is commonly identified to be related with insecticide resistant issues closely related to another detoxification gene, such as esterase and glutathione S-transferases [59,60].

As for POLD1, it was upregulated at every time points of PI stages, except 3 h and 144 h. Expression of POLD1 required by DNA polymerase delta to form DNA clamp with the proliferating cell nuclear antigen (PCNA) in a way to take over the synthesis leading and lagging strand from DNA polymerase [61,62,63]. Besides, the expression of this particular protein also has been known as a reaction due to DNA damage occurrence [64].

The Sec8 expressed by *M. anisopliae* was reported as one of the important subunit proteins that encodes together within EXOC4, the gene responsible for the exocyst complex [65]. Its role is by targeting exocytic vesicles from intercellular on the specific docking site on the plasma membrane [66]. Although the first report on its discovery in formation of the sec6/8 complex was described as a complex to specify the vesicle transportation to the restricted site on plasma membrane in yeast [67], it was also reported performing the same function and regulation in mammalian cells for vesicles delivery [68]. As Sec8 was reported as present at the intrakingdom level, therefore it is plausible that the presence of Sec8 in the total protein of *M. anisopliae* may perform similar function, which it also contributes to cell surface polarity in cell–cell adhesion [69,70] during conidial attachment on termites’ cuticle on early pathogenesis.

Several studies on heat shock proteins in *Metarhizium* had provided important information on its function: hsp70 was found involved exclusively within conidia responsible to host cuticle degradation, penetration of appressorium and protective processes [71], and hsp25 was observed overexpressed in *M. robertsii* during the extreme condition for survival in soil [72]. In general, heat shock proteins are known as stress proteins that display expressions in response to the stress condition [73]. It was reported related with the intercellular protein–protein interaction, serving the function as chaperone in assisting protein folding and transportation of the unfolded protein across the cell membrane [74,75]. It also was recorded in boosting conidia ability to survive under the stress environment [23].

Meanwhile, Catp is mainly involved in oxidoreduction activity. Previous studies have reported the increment in Catp expression could reduce the germination time and increases the fungus virulency during the infection process [76,77]. It is also involved in insect hydrocarbon catabolism when researchers found it exponentially expressed when changing the glucose with insect-like hydrocarbon under the in vitro condition [77]. In an observation carried out on *Beauvaria bassiana*, another potential entomopathogenic fungus, the removal of Catp had led to an increment on the fungus tolerability to encounter exposure of UV-B and oxidative stress during the germination level, but resulted decrement in its virulence as much as 50% in killing its insect host [78].

Lipoic acid plays an important role as a biological antioxidant in most organisms [79], thus its presence in *Metarhizium* may be related to assisting the survival of the fungus, preventing it from being oxidized in the host system during the germination and dissemination stage. Lias is known to be involved in synthesizing lipoic acid from octanoic acid within mitochondria, which is an essential metabolic gene for many organisms such as yeast or mammals [80,81,82]. The presence of lipoic acid in the living organism is vital as they act as a cofactor in enzyme complexes involved in central metabolism [80]. Discovery of lipoic acid synthetase in *Metarhizium* was first reported in *M. acridum*, which suggested the presence of lipoic acid in two subpathways: The protein lipoylation pathway and a part of protein modification via the endogenous pathway [83]. On the other hand, ATP-dependent RNA helicase DBP8 (spot 51, RNAh) was reported as one of the enzyme family involved in RNA metabolism and translation [84,85]. Although there is no literature about the regulation of RNA helicase during infection of *Metarhizium* on the insect host, yet several studies showed the importance of RNA helicase for in-host replication purposes when infecting certain hosts using different organisms, such as virus [86], bacteria [87,88], and fungi [86]. In another study, the disruption of RNAh in the pathogen *Borrelia burgdorferi* that is responsible for Lyme disease in humans, resulted to its inefficiency in infectivity and virulence [89]. Therefore, the expression of RNAh by *M. anisopliae* could also be related to its replication activity during dissemination and colonization in the host during the infection process, promoting continuous infectivity in the insect host.

The four significant proteins identified in termites, Nubp1, GTP-1p, POLD1, and Cyp6a13 showed the protein expression pattern more than the 20 × 10^3^ unit in the early stage of pathogenesis. The former three proteins displayed upregulated expression 1 h PI, which suggested that each protein holds an important role in the cellular signaling (Nubp1), immunity signaling (GTP-1p), and DNA damage (POLD1) (Figure 6). This could be caused by the activation of the host’s defense system when detecting the presence of foreign substances, such as the conidia of the entomopathogenic fungus, on the cuticles of the termite during 1 h PI [53]. The latter protein Cyp6a13, on the other hand, displayed upregulated expression at 3 h PI. It was suggested that the expression of this particular protein could be related to the activating metabolism of xenobiotics to encounter the infection by *M. anisopliae* during the survival mode [60].

Meanwhile, three out of five protein spots (Sec8, Hsp78, Lias); significance expressed protein by the fungus were observed to express as early at 1 h PI. These three protein spots have a special role in conidia attachment and survival during the early stage of infection on the termite’s host. The expression of Sec8 on 1 h PI may be due to the polarization of membrane infected termites in preparation of conidia attachment. This particular protein once again expressed at 6 h PI and decreased slowly at the following hour of PI. This may suggest that the requirement of this protein in cell adhesion and vesicles transportation required for release enzyme during germination are decreased following the successfulness of this pathogen to invade the termite’s host. Another protein, Hsp78 were kept expressed from the early stage until the final stage of infection. Presence of this protein at the early stage suggested that it was required for host cuticle degradation, penetration of appressorium, and protective processes [71]. The protein was continuously expressed throughout the whole process, maybe due to stress in response to the termite’s defense system when the pathogen is in contact during adhesion, germination, and dissemination [72]. Lias was observed with upregulated expression at the early stage of infection; between 1 and 12 h PI. Since Lias is an antioxidant and also a cofactor for enzyme complexes involved in central metabolism [79,80], it was suggested that this compound was released when the pathogen encountered the host’s immune system by releasing a large complex toxin compound such as destruxin [90].

The expression of the other two identified proteins, Catp and RNAh, showed different regulations throughout the infection process, whereby both proteins were highly expressed at 3 h and 24 h PI, respectively. The expression of Catp during that stage suggested that the presence of the preparation step in assisting chitinase to break down the termite’s exocuticles took place before 6 h PI, as it was reported that Catp was exponentially expressed when replacing the glucose with insect-like hydrocarbon during the in vitro condition [78]. On the other hand, RNAh was highly expressed at 24 h PI, maybe due to the reproduction of pathogen towards completing its life cycle, thus resulting in fungal dissemination occurring inside the host.

In this study, no recurring proteins from previous studies have been identified, either proteins related to the insect immunity such as lysozyme and prophenoloxidase [91], or proteins related to pathogenesis such as protease; subtilisin-like proteins (Pr1) and trypsin-like proteins (Pr2) [25,92]. This might be due to lower expression were displayed by these proteins, making them difficult to be detected using basic stains such as Coomassie blue [93,94]. Furthermore, the lack of protein-related studies on insects, particularly on termites, could also be one of the contributing factors towards lower and smaller useful protein database, thus imposing great challenges to this study. Providentially, differential in protein expression at different pathogenesis stages, from both organisms during the infection process determined in this study, corroborated with the previous study on the insect–pathogen interaction of the same pathogen but in a different host [95]. To provide more comprehensive information, different techniques and approaches, which are more sensitive and reliable, such as silver staining and liquid chromatography mass spectrometry (LC–MS) should be considered. These techniques are expected to yield higher resolutions in protein expression profiling; have the ability to detect lowly expressed and small-sized proteins, indirectly reducing the risk of losing unnoticed yet important proteins. Furthermore, it is advisable to cross-check with several protein databases to broaden the peptide mass fingerprint search in protein identification, in which this study utilized the genus *Metarhizium* and order Isopteran protein database from NCBI non-redundant (NCBInr) and Swiss-Prot. Therefore, by enhancing the technique and strategy in future studies, more information especially on the molecular interaction between this unique host–pathogen relationship could be understood.

## 5. Conclusions

We found that *M. anisopliae* had begun with a germ tube penetrating the *C. curvignathus* cuticles as early as 6 h PI. Effective colonization by *M. anisopliae* was found in infected termites attributed to an excess of blastospores in host hemocoel at 96 h PI. In the meantime, the host–pathogen interaction proteome offered insight into the major proteins involved in immunity of *C. curvignathus* (Nubp1, Cyp6a13, GTP1-1p, and POLD1) and *M. anisopliae* pathogenesis (Sec8, Hsp78, Catp, Lias, and RNAh) throughout the infection process.

In short, *M. anisopliae* may serve as a possibly infectious pathogen for termites, although there are instances in which termite immunity and sociobehavior are identified as apparent synergies with infection. However, based on the promising invasion capabilities seen in this study, the capacity of *M. anisopliae* to become a biological control agent for managing the termite population should also be regarded in future endeavors.

## Figures and Tables

**Table 1:** MALDI-TOF mass fingerprinting data of the 13 differentially expressed proteins isolated from *Metarhizium anisopliae*-infected *Coptotermes curvignathus*. Proteome changes in the infected termite were compared in a time-lined post-inoculation (PI) experiment to control proteins, extracted from 2D electrophoresis gels prior to identification.

Spot ID	Score		No. of Peptides Matched	Sequence Coverage (%)	Theoretical Mr/pI		Protein Identity	Metabolic Pathway	Cellular Location	Accession no. (NCBInr), (Organism)
	SWISS PROT	NCBInr			Mr (kDa)	pI				
21	44	331	11	26	42.72	6.15	Phosphotriesterase-related protein	Zinc-ion binding	Extracellular exosome	KDR19492 (*Zootermopsis nevadensis*)
31	34	387	6	13	58.90	8.63	Putative cytochrome P450 6a13	Monooxygenase	Membrane	KDR19800 (*Zootermopsis nevadensis*)
37	44	55.8	18	18	40.33	8.90	WD repeat- containing protein 5 isoform X1	Histone acetylation	Nuclear region	KDR13642 (*Zootermopsis nevadensis*)
40	53	761	7	26	41.79	5.30	Actin-5C	ATP-binding	Cytoskeleton	KDR24293 (*Zootermopsis nevadensis*)
45	55	626	17	27	67.28	6.04	Large subunit GTPase 1-like protein	GTP-binding GTPase activity	Membrane	KDR18965 (*Zootermopsis nevadensis*)
47	30	357	3	19	28.21	7.67	Proteasome subunit alpha type-7-like	Endopeptidase activity	Cytosol/nuclear region	KDR19303 (*Zootermopsis nevadensis*)
57	30	364	4	20	33.77	4.91	Cytosolic Fe-S cluster assembly factor Nubp1	ATP-binding	Cytosol	KDR12672 (*Zootermopsis nevadensis*)
60	43	229	10	19	110.62	6.50	Rho GTPase- activating protein 17	GTPase activation	Membrane	KDR10828 (*Zootermopsis nevadensis*)
65	44	162	26	21	116.44	8.71	DNA polymerase delta catalytic subunit	Nucleotide binding	Nuclear region	BAJ78756 (*Reticulitermes speratus*)
67	36	133	6	24	56.79	6.62	Maelstrom-like protein	n/a	n/a	KDR08911 (*Zootermopsis nevadensis*)
70	35	53.5	6	20	19.96	8.17	Exonuclease 3’-5’ domain-containing protein 2	Mature miRNA 3’-end processing	Nuclear region	KDR07728 (*Zootermopsis nevadensis*)
75	45	525	17	21	111.68	5.99	Protein diaphanous	Cytokinesis	Cytoskeleton	KDR17398 (*Zootermopsis nevadensis*)
83	41	325	20	10	221.97	5.14	Protein disabled	Synaptic vesicle endocytosis	Cytoplasm	KDR22018 (*Zootermopsis nevadensis*)

**Table 2:** MALDI-TOF mass fingerprinting data of the nine differentially expressed proteins isolated from *Metarhizium anisopliae* infecting *Coptotermes curvignathus*. Proteome changes in the infecting fungus were compared in a time-line post-inoculation (PI) experiment to control proteins, extracted from 2D electrophoresis gels prior to identification.

Spot ID	Score		No. of Peptides Matched	Sequence Coverage (%)	Theoretical Mr/pI		Protein Identity	Metabolic Pathway	Cellular Location	Accession no. (NCBInr), (Organism)
	SWISS PROT	NCBInr			Mr (kDa)	pI				
16	46	174	6	57	29.14	5.64	Methylthioribulose-1-phosphate dehydratase	L-methionine biosynthesis via salvage pathway	Cytoplasm	OAA48651 (*Metarhizium rileyi* RCEF 4871)
27	90	196	28	34	119.45	5.63	Sec8 exocyst complex component specific domain protein	Exocystosis	Exocyst	KID86834 (*Metarhizium guizhouense* ARSEF 977)
29	55	447	13	47	45.92	9.19	Lipoic acid synthetase	Lipoate biosynthetic process	Mitochondrion	XP_014580459 (*Metarhizium majus* ARSEF 297)
32	65	914	17	31	87.74	6.29	Heat shock protein 78	ATP-binding	Nuclear region	XP_014546332 (*Metarhizium brunneum* ARSEF 3297)
51	48	414	15	39	60.05	7.25	ATP-dependent RNA helicase DBP8	ATP-binding	Nuclear region/cytoplasm	OAA38096 (*Metarhizium rileyi* RCEF 4871)
87	48	434	13	36	53.00	6.06	ATP dependent RNA helicase DBP5	ATP-binding	Nuclear region/cytoplasm	XP_007808948 (*Metarhizium acridum* CQMa 102)
88	45	334	7	30	41.82	6.12	Methylthioribose-1-phosphate isomerase	L-methionine biosynthetic process	Nuclear region/cell projection	XP_014549852 (*Metarhizium brunneum* ARSEF 3297)
89	39	43.5	8	29	58.41	5.43	Defects-in-morphology protein 1-like	Nucleic acid phosphodiester bond hydrolysis	Nuclear region/cytosol	OAA48471 (*Metarhizium rileyi* RCEF 4871)
90	62	21.8	22	20	56.04	6.4	Peroxisomal catalase	Peroxidase/ oxidoreductase activity	Peroxisome	XP_007809457 (*Metarhizium acridum* CQMa 102)

## Data Availability

Data sharing not applicable.
